# Chronic diseases and mortality among hospitalised COVID-19 patients at Bafoussam Regional Hospital in the West region of Cameroon

**DOI:** 10.1371/journal.pgph.0001572

**Published:** 2023-02-23

**Authors:** Imelda Sonia Nzinnou Mbiaketcha, Collins Buh Nkum, Ketina Hirma Tchio-Nighie, Iliasou Njoudap Mfopou, Francois Nguegoue Tchokouaha, Jérôme Ateudjieu

**Affiliations:** 1 Department of Public Health, Faculty of Medicine and Pharmaceutical Sciences, University of Dschang Cameroon, Dschang, Cameroun; 2 Department of Health Research, M.A. SANTE (Meilleur Acces aux Soins de Sante), Yaounde, Cameroon; 3 Department of COVID-19 Management Bafoussam Regional Hospital, Bafoussam, Cameroon; 4 Department of Internal Medicine, Bafoussam Regional Hospital Center, Bafoussam, Cameroun; 5 Division of Health Operations Research, Cameroon Ministry of Public, Yaounde, Cameroon; New York University Grossman School of Medicine, UNITED STATES

## Abstract

Reducing mortality among COVID-19 cases is a major challenge for most health systems worldwide. Estimating the risk of preexisting comorbidities on COVID-19 mortality may promote the importance of targeting at-risk populations to improve survival through primary and secondary prevention. This study was conducted to explore the contribution of exposure to some chronic diseases on the mortality of COVID-19. This was a case control study. The data were collected from the records of all patients hospitalised at Bafoussam Regional Hospital (BRH) from March 2020 to December 2021. A grid was used to extract data on patient history, case management and outcome of hospitalised patients. We estimated the frequency of each common chronic disease and assessed the association between suffering from all and each chronic disease (Diabetes or/and Hypertension, immunodeficiency condition, obesity, tuberculosis, chronic kidney disease) and fatal outcome of hospitalised patients by estimating crude and adjusted odd ratios and their corresponding 95% confidence intervals (CI) using time to symptom onset and hospital admission up to three days, age range 65 years and above, health professional worker and married status as confounder’s factors. Of 645 included patients, 120(20.23%) deaths were recorded. Among these 645 patients, 262(40.62%) were males, 128(19.84%) aged 65 years and above. The mean length of stay was 11.07. On admission, 204 (31.62%) patients presented at least one chronic disease. The most common chronic disease were hypertension (HBP) 73(11.32%), followed by diabetes + HBP 62 (9.61%), by diabetes 55(8.53%) and Immunodeficiency condition 14(2.17%). Diabetes and Diabetes + HBP were associated with a higher risk of death respectively aOR = 2.71[95%CI = 1.19–6.18] and aOR = 2.07[95% CI = 1.01–4.23] but HBP did not significantly increased the risk of death. These results suggest that health authorities should prioritize these specific group to adopt primary and secondary preventive interventions against SARS-CoV-2 infection.

## Introduction

The COVID-19 pandemic has become a global public health threat, endangering the health and well-being of populations [1, 2]. The response to this pandemic requires the identification and control of factors that increase its attack and case fatality rates (CFR) [3, 4]. Since the onset of the COVID-19 pandemic, several studies have been conducted to identify the determinants of its transmission, morbidity, and mortality. Poverty, low level of education, fragility of the health system, overcrowding in urban areas and climate have been identified as factors that increase the risk of transmission of the virus [5–9]. COVID-19 patient with diabetes, obesity, chronic obstructive pulmonary disease (COPD), cardiovascular disease (CVD), hypertension, malignancies, Human Immunodeficiency (HIV) virus and other comorbidities may develop a life-threatening condition. Comorbidities lead the COVID-19 patient into an infectious vicious cycle and are substantially associated with significant morbidity and mortality. Comorbid individuals require vigilant preventive measures and careful management [10]. In the current state of knowledge COVID-19 infection in patients aged age 65 years and above, male, and non-communicable pathologies has been found to contribute in inducing complication of COVID-19 and to increase the probability of death [11–13]. Furthermore, some studies have reported that healthcare workers are more likely to develop COVID-19 [14, 15], to be hospitalised and to die [16] despite having pre-existing conditions.

These determinants necessary for effective monitoring of pandemic’s progress may vary across countries or settings and need to be considered to tailor the response to the realities of the pandemic in each setting.

In Cameroon, the epidemic began in March 2020. The response consisted of government measures such as closing all borders, closing bars and restaurants after 6:00 p.m., restricting people on public transport, closing schools and universities, and mandating the wearing of masks in public places [17]. These initial strict government measures were quickly relaxed [18, 19] to including the establishment of specialized centers for the management of COVID-19 cases, the decentralization of interventions, the integration of operational research, the availability of rapid diagnostic tests that improve screening capabilities, and the establishment of a national surveillance platform to track circulating SARS-CoV-2 have helped limit disease transmission. In addition, the implementation of a vaccination program since April 2021, despite public reluctance, would strengthen the control of the epidemic. Despite these primary and secondary preventive measures taken at individual and collective levels, COVID-19 is still characterized by a relatively high CFR of 1,7% in Cameroon as on the 31^st^ December 2021 [20]. To the best of our knowledge, many studies have been conducted on the effects of comorbidities on the risk of death in COVID-19 in other countries [10–13] but none of them reflect the situation of Cameroon where the epidemiology of disease depends on the physical, financial and acceptability of health care services to populations [21, 22]. This lack of information in Cameroon explains why this issue was investigated in the present study.

This study was conducted to assess the distribution of chronic disease cases and deaths, among COVID-19 hospitalised and death patients at the Bafoussam Regional Hospital (BRH), the intermediate care level in one of most affected regions of Cameroon and to determine if any of these conditions increases the risk of mortality among COVID-19 patients.

The findings are expected to be shared with health personnel and authorities in charge and interested scientists to contribute to the review of policy and research needs for a better response to COVID-19 in Cameroon and other developing countries with similar parameters.

## Materials and methods

### Operational definition

#### Diabetes

Blood glucose level, at any time of the day, is higher than 2 g/l in the presence of symptoms such as polyuria, polydipsia, constant hunger, weight loss, visual impairment and fatigue; the fasting blood glucose level is ≥ 1.26 g/l, tested twice in the absence of symptoms. In order to confirm the result of the fasting blood glucose test, a second blood test is performed.

#### HBP: Hypertension

(High blood pressure-HBP) is the permanent elevation of blood pressure (BP) above 140mm Hg for systolic pressure and 90 mm Hg for diastolic blood pressure.

#### Immunodeficiency condition

Detection in blood sample of antibodies against human immunodeficiency virus (HIV).

#### Obesity

Body mass index (BMI) ≥30, calculated as weight divided by the square of height, expressed in kg/m2.

#### Tuberculosis

Presence of *Mycobacterium tuberculosis* determined by microscopic examination, bacteriological examination of the sputum, broncho alveolar lavage and/or tests of gene amplification.

#### Chronic kidney disease

Is a long-standing and progressive deterioration of kidney function. The diagnosis is based on laboratory tests of the renal function using estimated glomerular filtration rate [eGFR] < 60 mL/min/1.73 m2 or urine albumin to creatinine ratio [ACR] ≥ 30 mg/g).

**COVID-19** (Coronavirus disease 2019): presence of SARS-CoV-2 virus detected by reverse transcription polymerase chain reaction (RT-PCR) or by a rapid diagnostic test (BIO RAD RDT COVID test kit).

### Ethical considerations

The present study is aim at providing information on factors that increase case fatality in hospitalised COVID-19 cases, which will allow targeting of primary and secondary interventions to help reduce case fatality in these groups. The protocol was evaluated and approved by the Cameroon National Ethics Committee for Human Health Research (N°2022/04/110/CE/CNERSH/SP). In addition, administrative authorization was obtained from the Director of BRH to collect data from the patient records of the COVID-19 case management center. The information collected was coded and anonymous, and access was restricted to researchers involved in the data collection.

### Study design

This was a case control study. Data were collected from the files of COVID-19 patients hospitalised at the BRH from March 2020 to December 2021. The descriptive part allocated to distribute chronic disease cases and deaths among COVID-19 hospitalised and death patients and case control component assessed the effect of presenting each chronic disease as a comorbidity on the risk of COVID-19 mortality.

The cases were the deceased patients and were matched to controls who were 02 recovered patients of the same sex. The exposure was defined as the presence of diabetes and/or HBP, immunodeficiency condition, obesity, tuberculosis, chronic kidney disease. The association between these exposures and the occurrence of COVID-19 death was estimated by calculating crude odd ratio (OR) and adjusted OR with age 65-year and above, married status, time from symptom onset to hospital admission of three days or more, and health professional.

### Study site and period

The study was conducted at the COVID-19 Case Management Center at the BRH, the best technically equipped hospital in the western region for the management of severe COVID-19. This reference center was selected and has, among other missions, the management of symptomatic and eligible COVID-19 cases referred by the 20 health Districts of the region since the beginning of the pandemic in March 2020. Data’s were collected from March 2020 to December 2021.

### Study population

Was included for the descriptive component of our study any patient who was tested positive for SARS-CoV-2 either by a reverse transcription polymerase chain reaction (RT-PCR) or a rapid diagnosis test (RDT) and was hospitalised at the COVID-19 case management unit at the BRH.

### Sampling and sample size

For the descriptive component, all 645 patients hospitalised at the BRH for the period of the study were included. For the case-control component, same-sex controls were randomly matched to cases. The sample size estimated was 120 cases and 240 controls, assuming a study power of 90%, level of significance of 5%, that the risk of death will be about 50% and an expected odd ratio of at least 2, 5 between exposures and death. The estimation was guided by the WHO (World Health Organization) manual for sample size determination in health practices [23].

### Inclusion and exclusion criteria for cases and controls

#### Selection of cases (deaths)

We included patients who tested positive for SARS-CoV-2, were hospitalised and died from COVID-19 and excluded patients who were transferred, escaped, had undocumented exposure or treatment outcome or had incomplete data.

#### Selection of controls (recovered)

We included patients who tested positive for SARS-CoV-2, were hospitalised and recovered of COVID-19 and excluded patients who were transferred, escaped, whose exposure or treatment outcome was not documented or whose data were incomplete.

**Criteria for matching** cases to controls: were randomly matched to 1 case 2 controls of same-sex and who recovered after hospitalisation.

### Data collection tools

Data were collected from patient records using a data collection grid in Microsoft Excel developed by the study team and pretested in a district hospital in the western region. The main variables collected were chronic diseases of hospitalised cases, admission symptoms, outcome (death or recovered) temporal location and socio-demographic characteristics.

### Data management

Data were reviewed after completing the data collection grid in Microsoft Excel to detect any omissions or confusing observations. These data were compared to the existing hospital database and any corrections were made. The data collected was processed daily and cross-cheeked by a second person to detect and process errors.

### Data analysis

For descriptive analyses, proportions and means were estimated. Crude and adjusted odds ratios were used to estimate the associations between the presence of diabetes and/or HBP, immunodeficiency condition, obesity, tuberculosis, chronic kidney disease and death of COVID-19. Chi-square tests were used to compare the characteristics of the case and control groups and the characteristics that differed were included as adjustments: health professional, age 65 years and above, married status and time from symptom onset to hospital admission for three days or more. Analyses were performed using IBM SPSS version 23 (IBM Corporation, IBM SPSS Statistics for Windows, Version 23.0. Armonk, New York.) and Microsoft 2019 Excel software (Microsoft Corporation, Microsoft Excel. 2019) and p-value < 0.05 were considered significant.

## Results

**Fig 1:**
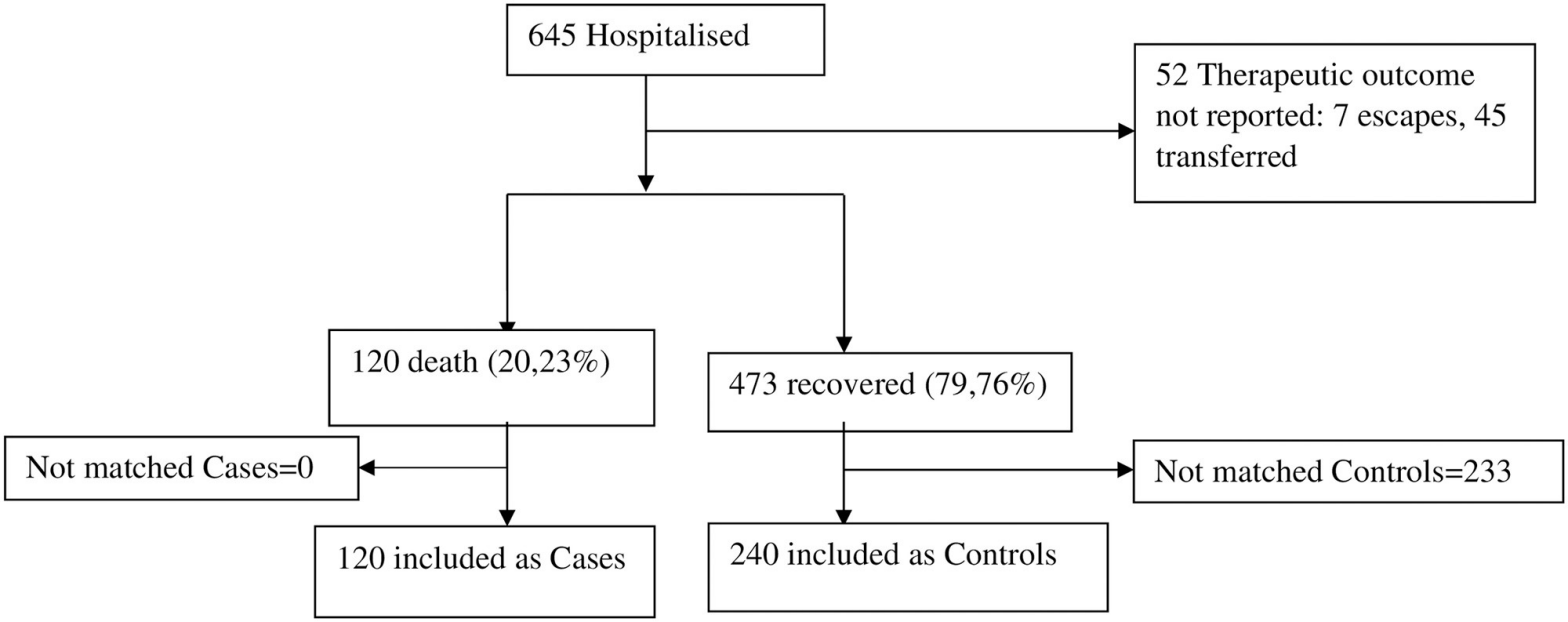
Flow chart of the study.

From the onset of the pandemic in March 2020 to December 2021, 645 COVID-19 patients were hospitalised at the COVID-19 Case Management Center at the BRH. Only 593 patients met the inclusion criteria, of which 120 (20.23%) died and 473 (79.76%) recovered. Of these 593, 360 were included in the case-control arm: 120 included as cases and 240 as controls. The number of patients included at each stage of the study are shown in Fig 1.

### Study population characteristics

A total of 645 patients were hospitalised at BRH from March 2020 to December 2021. The median age was 43 years (interquartile range 31–61). Of all hospitalised patients 262 (40.62%) were male and 128(19, 84%) were aged 65 and above. Almost all patients were married: 591(91.63%). Health professional were represented as 85 (13.18%) (Table 1).

**Table 1:** Socio-demographic characteristics of the COVID-19 confirmed cases admitted to BRH.

Modalities	Frequency (N)	Proportion (%)
**Profession**		
Self-employment	10	1,55
Farmer/trader	26	4,03
Electro technician	23	3,57
Employee	10	1,55
Student	41	6,36
Civil servant	56	8,68
Housekeeper	102	15,81
Not documented	225	34,88
Health professional	85	13,18
Religious	5	0,78
Pensioner	62	9,61
**Sex**		
Female	383	59,38
Male	262	40,62
**Marital status**		
Single	37	5,74
Married	591	91,63
Not documented	13	2,02
Widow	4	0,62
**Age range**		
[15–49]	385	59,69
[50–64]	132	20,47
≥65	128	19,84

**Fig 2:**
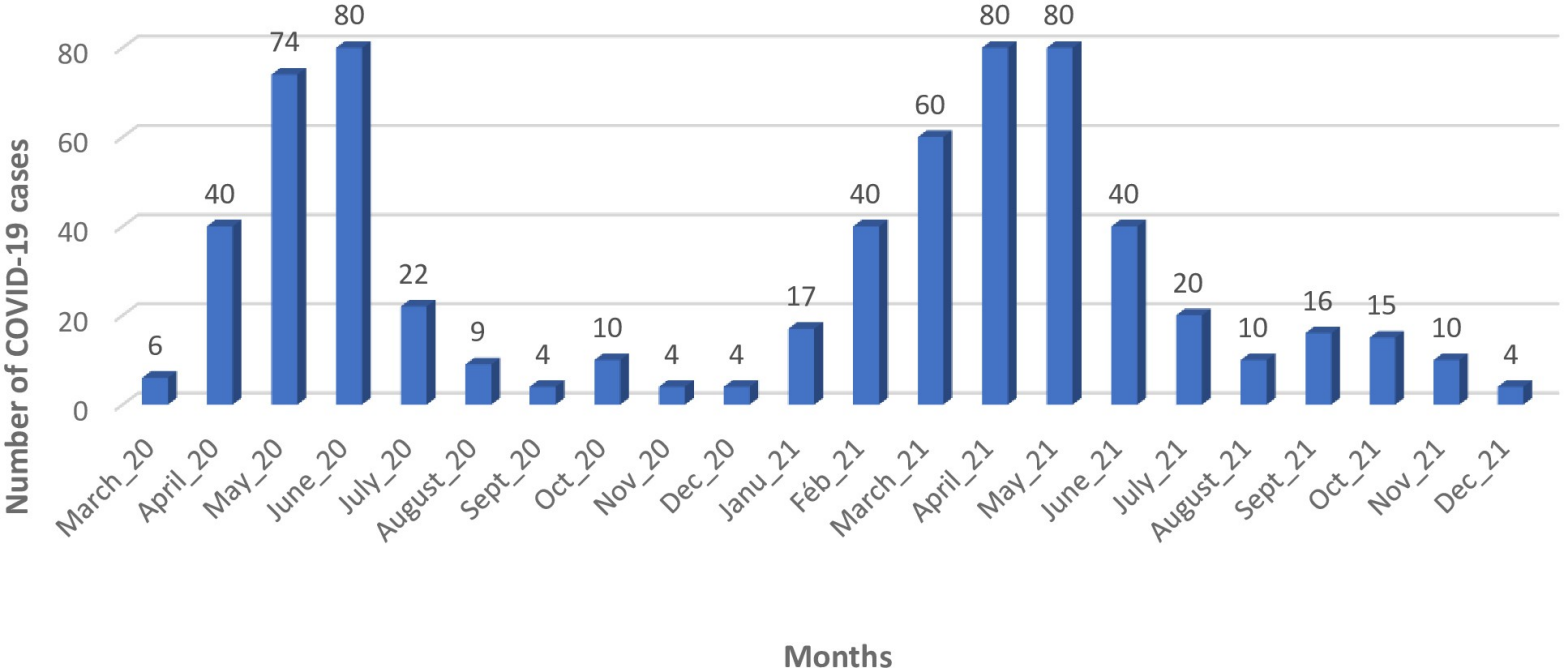
Temporal distribution of COVID-19 confirmed cases: Hospitalised cases and deaths. March 2020-December 2021.

**The temporal distribution of COVID-19 confirmed cases admitted to BRH** is shown Fig 2. Two peaks of absolute increase in cases are noted from May to June 2020 and from April to May 2021.

**Fig 3:**
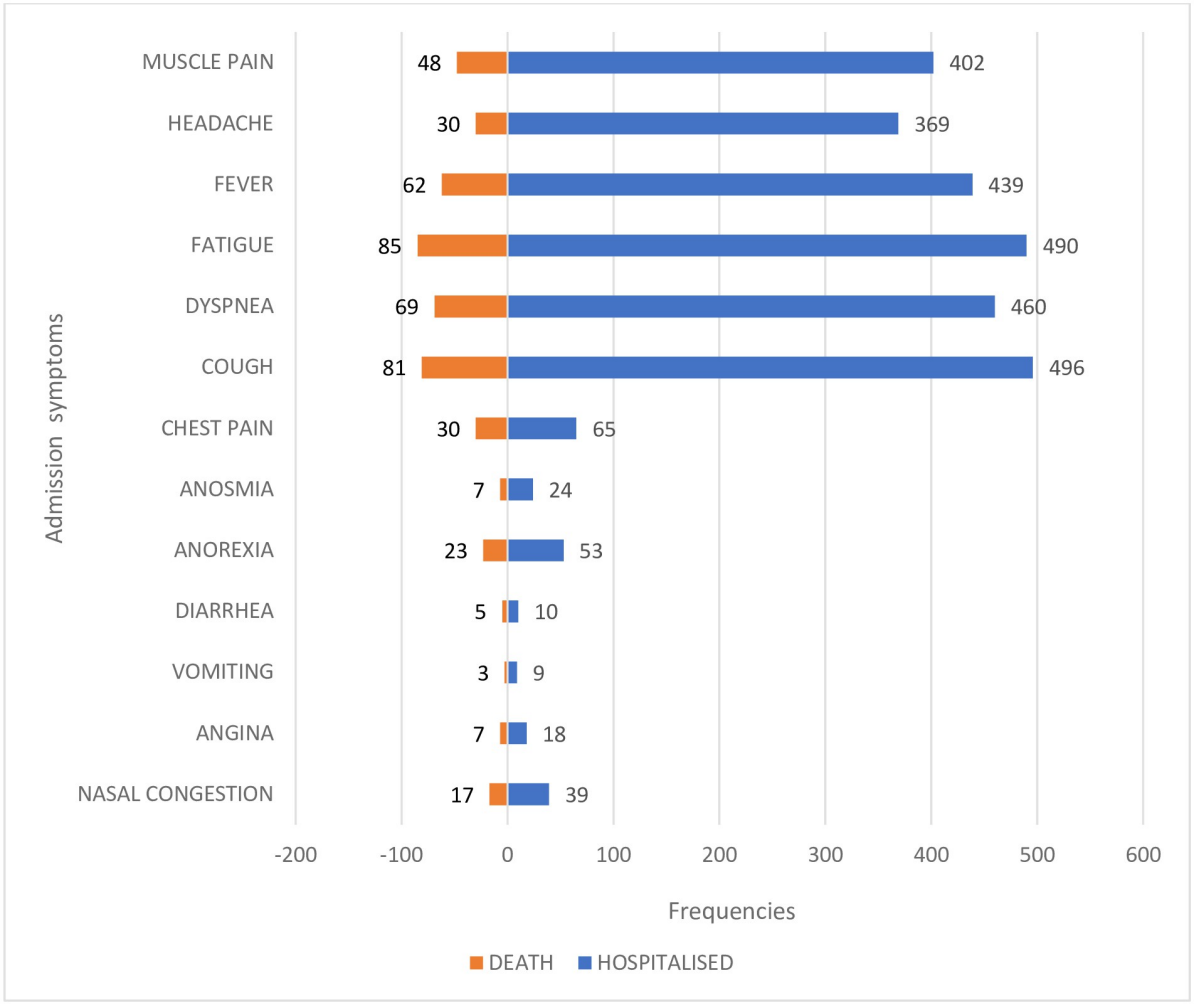
Distribution of COVID-19 confirmed cases and deaths according to symptoms on admission.

**The symptoms of COVID-19 confirmed cases on admission** are shown in Fig 3. The most frequent symptoms were cough (76.86%), fatigue (75.97%) and dyspnea (71.32%).

**Fig 4:**
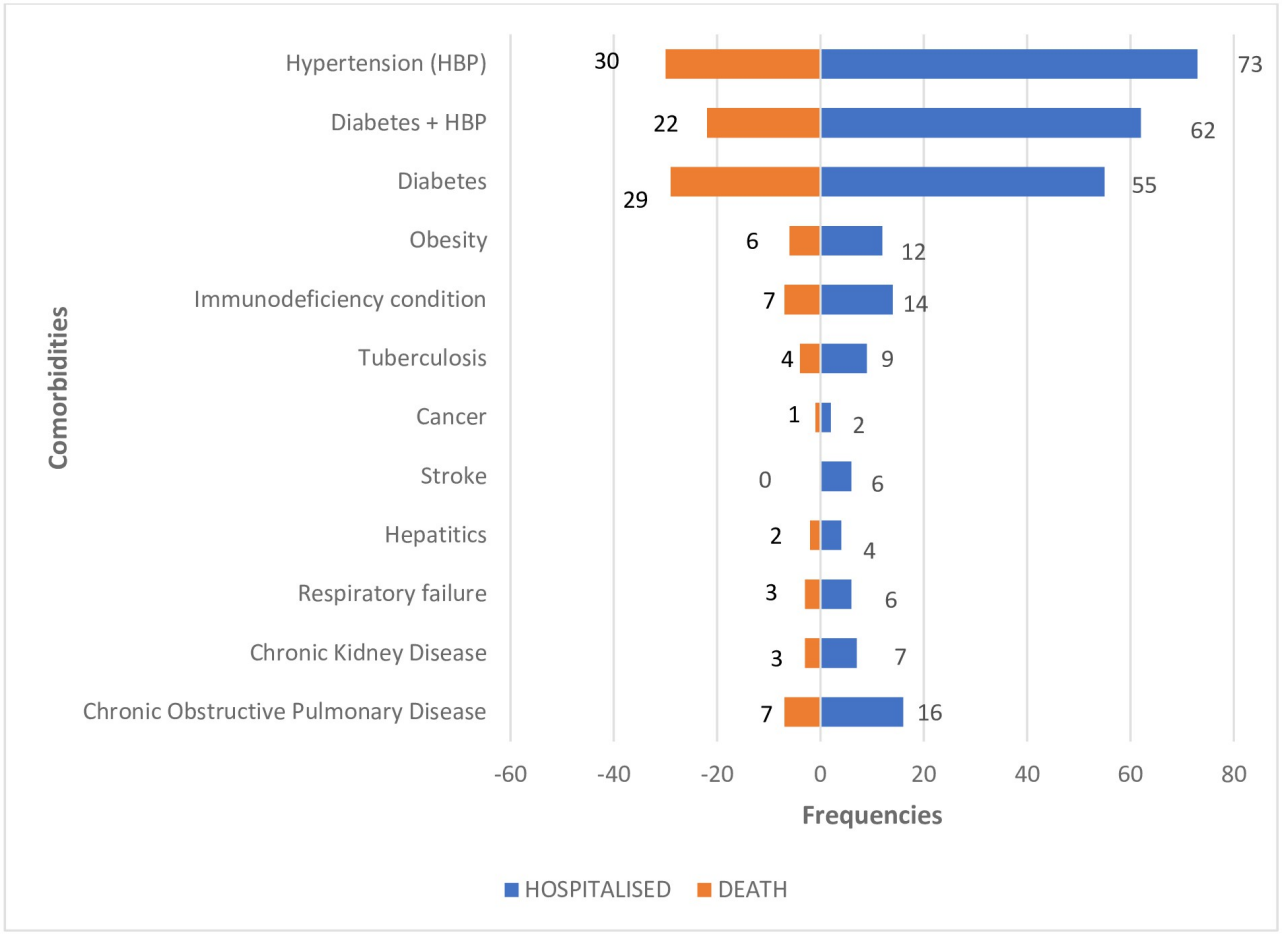
Distribution of COVID-19 confirmed hospitalised cases and deaths according to comorbidities.

**The most common comorbidities among COVID-19 confirmed patients hospitalised** at BRH were diabetes, hypertension, diabetes + hypertension and HIV positivity Fig 4.

### Characteristics of cases (Deaths) and controls (recovered) of COVID-19

A total of 360 patients were included in the case-control arm, 120 cases (deaths) and 240 controls (recoveries). The reported Chi-square shows that these characteristics are significantly different with respect to time to symptom onset and hospital admission up to three days, age range 65 years and above, health professional worker and married status. These characteristics being significantly different in cases and controls were considered confounding factors for this study. The comparison of socio-demographic characteristics of cases and controls are shows (Table 2).

**Table 2:** Comparison of case and control characteristics.

Variables	Therapeutical evolution			
	Cases n (%)	Controls n (%)	Chi2	p-value
**Age range**				
≥65	68(56,67)	35(14,58)	69,36	**0,000** *
<65	52(43,33)	205(85,42)		
**Symptom admission delay**				
>3	87(73,11)	99(41,6)	31,56	**0,000** *
< = 3	32(26,89)	139(58,4)		
**Marital status**				
Married	110(96,49)	209(88,56)	5,99	**0,014** *
Single/widow	4(3,51)	27(11,44)		
**Professions**				
Housekeeper	27(23,08)	31(17,03)	5,46	0,19
Health professional	17(14,53)	56(30,77)	10,17	**0,001** *

*Significant p-Value<0,05

### Association between chronic diseases and mortality of COVID-19 cases

In univariate logistic regression of each of the chronic disease with the therapeutic outcome death/recovered, it was found that diabetes, diabetes +Hypertension, obesity and immunodeficiency condition were significantly associated with the occurrence of COVID-19 mortality. However, after adjustment of each of these variables, only diabetes, diabetes + Hypertension and immunodeficiency condition remained significantly associated with the occurrence of COVID-19 death: diabetes significantly increased the risk of dying from COVID-19 by 2.71, diabetes and Hypertension significantly increase the risk of dying from COVID-19 by 2.07 and immunodeficiency condition increases the risk of dying from COVID-19 by 7.21 **(Table 3)**.

**Table 3:** Crude and adjusted association estimates.

Variables		Death (%)	Recovered (%)	Crude Association			Adjusted Association		
				cOR	95%CI	p-Value	aOR	95%CI	p-Value
Diabetes	Yes	29(24,17)	12(5,00)	6,05	2,96–12,38	0,000*	2,71	1,19–6,18	0,017*
	No	91(75,83)	228(95,00)						
Diabetes + HBP	Yes	30(25,00)	18(7,50)	4,11	2,18–7,74	0,000*	2,07	1,01–4,23	0,04*
	No	90(75,00)	222(92,50)						
Hypertension	Yes	22(18,33)	32(13,33)	1,45	0,80–2,64	0,21			
	No	98(81,67)	208(86,67)						
immunodeficiency condition	Yes	7(5,83)	4(1,67)	3,64	1,04–12,71	0,03*	7,21	1,49–34,7	0,013*
	No	113(94,17)	236(98,33)						
Obesity	Yes	6(5,00)	3(1,25)	4,15	1,02–16,09	0,03*	5,34	0,92–30,8	0,06
	No	114(95,00)	237(98,75)						
Tuberculosis	Yes	4(3,33)	2(0,83)	4,09	0,73–22,65	0,1			
	No	116(96,67)	238(99,17)						
Chronic Kidney Disease	Yes	3(2,50)	2(0,83)	3,05	0,5–18,4	0,2			
	No	117(97,50)	238(99,17)						

cOR, crude odds ratio; aOR, adjusted odds ratio, CI, Confidence interval,

*Significant p-Value<0,05; HBP, high blood pressure, HIV, human immunodeficiency virus.

## Discussion

This study was conducted to assess the contribution of chronic disease on the mortality of COVID-19 hospitalised cases in a hospital offering an intermediate level of care in Cameroon.

A total of 120 (20.23%) deaths were recorded among included hospitalised COVID-19 patients. Being diabetic, diabetics and hypertensive, or having immunodeficiency condition was found to be associated with a higher risk of death while being hypertensive did not significantly increase the risk of death among hospitalised COVID-19 patients.

The present study revealed a significant association between the presence of diabetes and COVID-19 mortality; crude OR = 6.05 [95% CI = 2.96–12.38] and adjusted OR = 2.71 [95% CI = 1.19–6.18]; this association was found both in a retrospective cohort study and in an analysis of global data from China in 2020 [24, 25] with a RR = 3.64 [95% CI = 1.09–12.21] and RR = 1.59 [95% CI = 1.03–2.45] respectively. Similar results were reported in other studies [26–30]. None of the data we have collected does not allow us to explain this association, but the plausible hypothesis could be that the vulnerability of these patients can be explained by the presence of comorbidities, notably cardiovascular disease [31]; and by a weakened immune response to the infection [32]. The data suggest that the increased disease severity observed in diabetes is likely due to a dysregulated immune response because of increased expression of ACE-2 (SARS-CoV-2 receptor) in diabetics; this may promote increased cellular binding to SARS-CoV-2. The virus is known to use ACE-2 receptors, which are found on the surface of host cells, to enter the cell [33]. High levels of ACE-2 receptors have been shown to be associated with diabetes, which may predispose diabetics to SARS-CoV-2 infection. A dysregulated immune response with increased ACE-2 receptors may lead to increased lung inflammation and lower insulin levels. The easy entry of the virus leads to a life-threatening situation for diabetic patients. Also, impaired T-cell function and high levels of interleukin-6 (IL-6) also play a decisive role in the development of COVID-19 disease in diabetics [34]. In addition, COVID-19 tends to progress in a high glucose environment, and fluctuations in blood glucose levels can compromise the immune system and make the viral infection more difficult to treat and longer lasting [35].

Although the sample size was small, this study was able to show that immunodeficiency condition was associated with a 3-fold increased risk of death from COVID-19: crude OR = 3.64 [95% CI = 1.04–12.71]. Till date, no available comparable study has been published in Cameroon. Our results are similar to those of a retrospective cohort study comparing the risk of death from COVID-19 in people living with and without HIV in the OpenSAFELY trial in England [36, 37], and different from those of other studies in other countries [37, 38]. People with immunodeficiency condition are at high risk of developing COVID-19 disease due to their weakened immune system.

Obesity was not a risk factor for COVID-19 in this study after adjustment for age 65 years and above, time from first symptom to hospital admission, health professional and married status (crude OR = 4.15 [95% CI = 1.02–16.09] and adjusted OR = 5.34 [95% CI = 0.92–30.08]); this result is similar to that reported in China [39]. This difference could be due to the difference in population, sample size and methodology. Furthermore, adjustment for intermediate factors may reduce the relationship between obesity and severity in COVID-19. Nevertheless, several other studies have found that a high body mass index (BMI) is a risk factor for the severity of COVID-19 [40, 41]. Indeed, obesity is generally associated with impaired lipid synthesis and clearance, which can trigger or aggravate inflammation and lung damage. It has been shown that for viral entry into the host cell, SARS-CoV-2 utilizes angiotensin converting enzyme 2 (ACE-2) receptors present on cells; diet-induced obesity showed a significant increase in ACE-2 expression in the lungs [42]. Further studies are needed to explore this relationship.

This study found no association between the presence of HBP (OR = 1.45, 95% CI 0.80–2.64) and the occurrence of COVID-19 mortality. This trend varied between studies. A cohort study of electronic data from patients in England showed that HBP was not a risk factor for COVID-19 mortality after adjusting for age, sex, and ethnic group (OR = 0.89 95% CI = 0.85–0.93) [43]. Similarly, in Italy, after a multicenter cross-sectional study involving 26 hospitals contacted by the network of the Italian Society of HBP in 13 regions in 2020, it was shown that HBP was not associated with the occurrence of death from COVID-19 after adjustment for age and sex (p-value = 0.944) [44]. Some studies found no association between the presence of HBP and the occurrence of mortality to COVID-19. However, other studies reported a significant association between the two [11, 28, 30, 45–47], These results may be explained by the difference in the population, the difference in our sample sizes. The data suggest that the use of different antihypertensive treatments in the population plays an important role: the risk of developing a severe form of COVID-19 was lower in patients treated with ACE inhibitors [45, 48]. Additional large-scale studies are needed to explore the causal relationships underlying the observed associations between HBP and mortality in COVID-19 and controlling for possible confounders, including age and various morbid conditions.

Interpretation and use of these results must take into account limitations such as low completeness of data, lack of standardization of data in records, poor completion of records, small sample size and difficulties in deciphering data in records; the study was conducted at a single site with limited external validity; many patients who tested positive for COVID-19 and died on admission to the management service were not included in our study; Also, we considered infectious pulmonary diseases (tuberculosis) and not pulmonary diseases (COPD) as comorbidity. Further studies are needed in the African context to clarify the consistency of the results.

## Conclusion

The overall objective was to assess the contribution of chronic disease to COVID-19 mortality at BRH. Approximately 18.60% of COVID-19 patients at that time died in this intermediate care hospital in Cameroon. Being diabetic, diabetics and hypertensive, or having immunodeficiency condition was found to be associated with a higher risk of death while being hypertensive did not significantly increase the risk of death among hospitalised COVID-19 patients. We can conclude that patients presenting some comorbidities are exposed to a relatively higher of risk of COVID-19 mortality.

This study allows to improve COVID-19 management, health professionals should better understand the chronic conditions that make patients more vulnerable to develop a severe complication and death due to COVID-19, to upgrade the management of COVID-19 patients with comorbidities, as opposed to patients without comorbidities, in order to control the danger of death. People with comorbidities should take vigorous preventive measures to protect themselves during the pandemic. The use of the COVID-19 vaccine would protect patients with comorbidities and they should be vaccinated as a priority. We recommend to the health authorities to prioritize diabetics, diabetics and hypertensive patients for primary and secondary prevention of COVID-19 cases, to validate appropriate therapeutic protocols to reduce morbidity and mortality; to improve and evaluate primary prevention; to the scientific Community we recommend to repeat the study prospectively with a large sample size to assess accurate associations between hypertension, obesity and morbidity-mortality in COVID-19.

## Supporting information

S1 Data This is the dataset used in the analysis. (XLSX)
